# The cysteine protease dipeptidyl aminopeptidase 3 does not contribute to egress of *Plasmodium falciparum* from host red blood cells

**DOI:** 10.1371/journal.pone.0193538

**Published:** 2018-03-06

**Authors:** Sreejoyee Ghosh, Scott A. Chisholm, Madeline Dans, Asha Lakkavaram, Kit Kennedy, Stuart A. Ralph, Natalie A. Counihan, Tania F. de Koning-Ward

**Affiliations:** 1 School of Medicine, Deakin University, Waurn Ponds, Victoria, Australia; 2 Department of Biochemistry and Molecular Biology, Bio21 Molecular Science and Biotechnology Institute, The University of Melbourne, Parkville, Victoria, Australia; Ehime Daigaku, JAPAN

## Abstract

The ability of *Plasmodium* parasites to egress from their host red blood cell is critical for the amplification of these parasites in the blood. Previous forward chemical genetic approaches have implicated the subtilisin-like protease (SUB1) and the cysteine protease dipeptidyl aminopeptidase 3 (DPAP3) as key players in egress, with the final step of SUB1 maturation thought to be due to the activity of DPAP3. In this study, we have utilized a reverse genetics approach to engineer transgenic *Plasmodium falciparum* parasites in which *dpap3* expression can be conditionally regulated using the *glmS* ribozyme based RNA-degrading system. We show that DPAP3, which is expressed in schizont stages and merozoites and localizes to organelles distinct from the micronemes, rhoptries and dense granules, is not required for the trafficking of apical proteins or processing of SUB1 substrates, nor for parasite maturation and egress from red blood cells. Thus, our findings argue against a role for DPAP3 in parasite egress and indicate that the phenotypes observed with DPAP3 inhibitors are due to off-target effects.

## Introduction

*Plasmodium falciparum*, an apicomplexan parasite, is one of the leading causes of global mortality and morbidity arising from an infectious disease [1]. The clinical symptoms of malaria consist of cyclical waves of fevers and chills, which coincide with the synchronous rupture of infected red blood cells (RBCs). This results in the release of parasite progeny termed merozoites that are capable of invading and proliferating within new host RBCs. Significant efforts have been made to understand the process by which *P*. *falciparum* invade RBCs (reviewed in [2,3]), but until recently our knowledge of how these intracellular parasites mediate their release from their host cell has been extremely limited [4–6]. Since the rupture of infected RBCs is critical for the propagation of the parasite, understanding how parasites mediate egress from the RBC is important as it may lead to the identification of new strategies to block parasite amplification within the blood. Certainly, the discovery of new anti-malarial drug targets is an urgent priority given the emergence of *P*. *falciparum* parasites resistant to the recommended artemisinin combination therapies [7] that threaten the gains that have been made in reducing the global burden of malaria in recent times.

While egress is known to be mediated by a highly regulated protease cascade [8], mechanistic insight into this process has been lacking. Forward chemical genetic approaches have implicated the proteases subtilisin 1 (SUB1) and dipeptidyl aminopeptidase 3 (DPAP3) as key players in egress [9,10]. SUB1 is one of three subtilisin-like proteases found in *P*. *falciparum* and is located in exonemes, a subcellular secretory organelle distinct from the other apical organelles (rhoptries, micronemes and dense granules) of the parasite [9]. Pharmacological blockade of SUB1 with the compound MRT12113 or JCP104 prevents schizont rupture and merozoite invasion. SUB1 acts on members of the serine repeat antigen (SERAs) family of papain-like proteins present in the parasitophorous vacuole (PV) in which the parasite replicates [9,10], resulting in destabilisation of the encasing PV membrane (PVM) [11,12]. SUB1 also plays an important role in the proteolytic maturation of the abundant merozoite surface protein 1 (MSP1), which is critical for interaction with the host RBC cytoskeleton to facilitate egress [5,13]. Bioinformatic and proteomic approaches have identified other proteins that are substrates or potential substrates of SUB1, including proteins that localise to the rhoptry bulb such as rhoptry associated protein 1 (RAP1), rhoptry associated membrane antigen (RAMA) and RhopH3 [14]. Thus SUB1, directly or through its action on its substrates, mediates development of invasive merozoites and their release from the host cell for the next round of invasion.

In addition to identifying the SUB1 inhibitor JCP104, the small molecule screen by Arastu-Kapur et al [10] identified a dipeptide vinyl sulfone inhibitor that specifically blocked parasite egress. With the aid of a biotin-labelled activity-based probe closely related to the cysteine protease inhibitors, the authors identified an unanticipated target—*P*. *falciparum* dipeptidyl aminopeptidase 3 (DPAP3), a parasite ortholog of human cathepsin C. DPAP3 is one of three dipeptidyl aminopeptidases encoded in the *Plasmodium* genome, which cleave dipeptides from the N termini of their substrates. DPAP1 is an essential food vacuole cysteine exopeptidase instrumental in haemoglobin digestion [15,16], while DPAP2 is a gametocyte stage-specific protease [17]. The localisation of DPAP3 has yet to be determined but development of a DPAP3-specific inhibitor (SAK1) that has minimal cross-reactivity with other proteases, produces a dose-dependent accumulation of mature, unruptured schizonts, providing further support for DPAP3 being critical for egress of asexual stage parasites [10]. SAK1 affects SUB1 maturation and SERA5 processing, which may be the mechanism through which DPAP3 contributes to parasite egress. Indeed, proteomic analysis revealed that 64% of the proteolytic events leading to egress occur downstream of DPAP3 activation [18]. This has led to the proposition that DPAP3 sits on top of the proteolytic cascade that leads to egress. Inhibition of DPAP3 also blocked the production and localisation of apical membrane antigen 1 (AMA1), a micronemal protein, and thus DPAP3 may regulate parasite egress by also regulating the maturation of secretory proteins required for this process. Arguing against a critical role for DPAP3 in egress, however, is the finding that DPAP3 can be knocked out in the rodent malaria species, *P*. *berghei* [19,20]. It is, therefore, important that the findings of SAK1 pharmacological blockade studies are validated in *P*. *falciparum* via other approaches to ensure SAK1 has no off target effects within the secretory pathway.

Thus to substantiate the role of DPAP3 in parasite egress and to assess whether this protease plays an important role in the maturation and trafficking of proteins to the apical organelles, we have examined the localisation of DPAP3 within the parasite and conditionally regulated its expression in *P*. *falciparum* using the *glmS* ribozyme based RNA-degrading system. We also observed that absence of DPAP3 does not affect the maturation of SUB1 substrates nor the ability to form healthy mature schizonts and egress, arguing against a role for DPAP3 in this process.

## Materials and methods

### Parasite culturing and creation of transgenic *P*. *falciparum* DPAP3-HAglmS line

**Fig 1:**
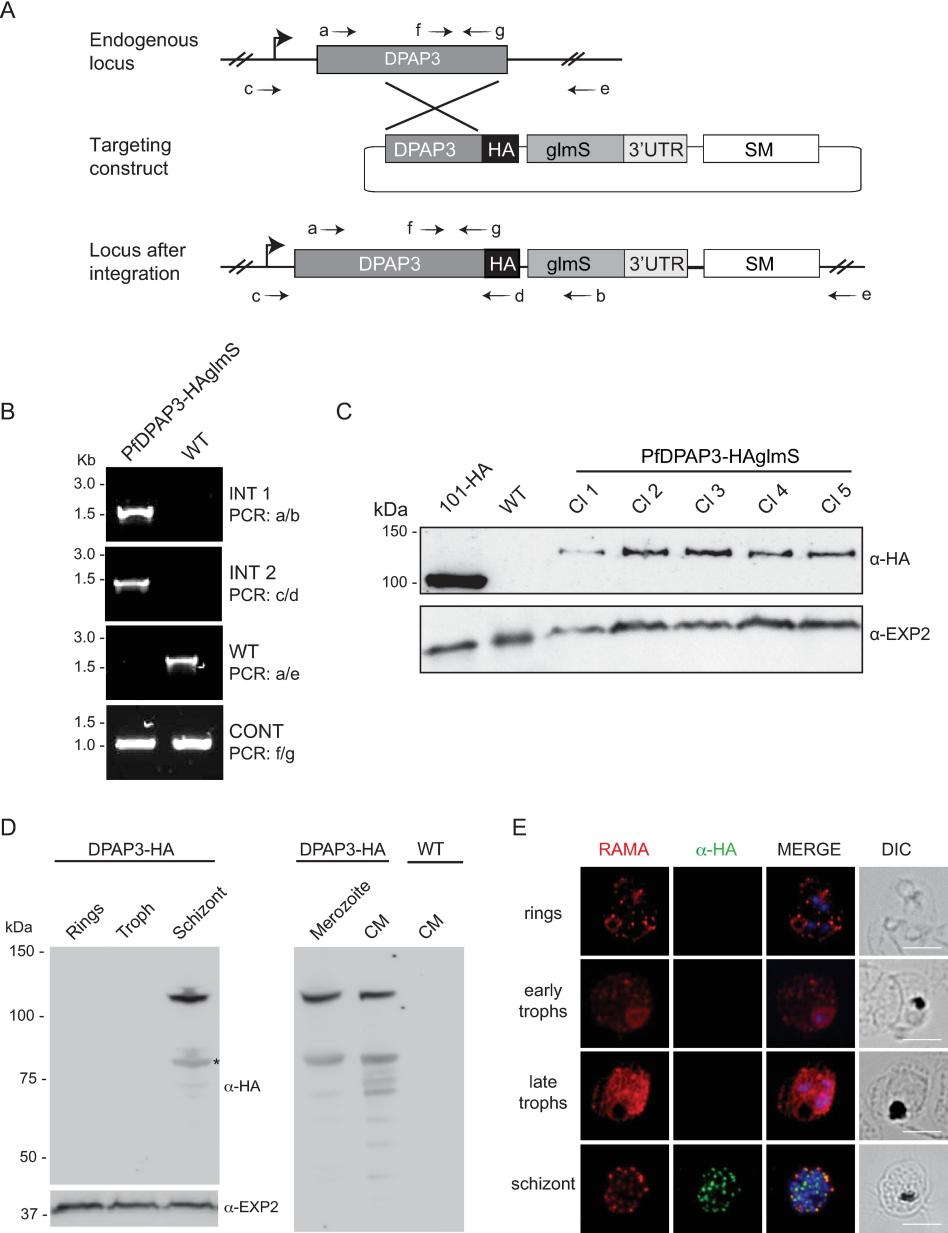
Creation of epitope tagged Pf*DPAP3* parasite line and analysis of DPAP3 expression. **A**. Overview of the *glmS* riboswitch system used to epitope tag as well as knock down DPAP3 in *P*. *falciparum*. The endogenous *dpap3* locus was modified to incorporate a triple haemagglutinin (HA) and streptavidin tag to the C-terminus of DPAP3, which is followed by the *P*. *berghei* DHFR-TS 3' untranslated region (3' UTR) that incorporates the ribozyme. In the absence of glucosamine (GlcN), translation proceeds as normal. However, addition of GlcN activates the ribozyme, leading to cleavage and subsequent degradation of the mRNA molecule. SM, selectable marker. The position of oligonucleotides (denoted a-f) used for diagnostic PCRs is indicated. **B.** Diagnostic PCR to determine that the expected integration event had occurred in the PfDPAP3-HAglmS line, with *P*. *falciparum* 3D7 parental line (WT) serving as a control. **C.** Western blot analysis of independent clones (Cl) of PfDPAP3-HAglmS line confirmed that DPAP3 had been successfully epitope tagged with HA. Lysates from 3D7 parasites in which HSP101 is HA-tagged (101-HA), served as a positive control for anti-HA labelling, while EXP2 served as a loading control. **D.** Western blot analysis showing DPAP3 is expressed in schizonts and merozoites and is also released into the culture medium. The 120 kDa band represents full-length DPAP3-HA, but a band of lower molecular weight ~85 kDa (indicated by an asterisks) was sometimes observed. EXP2 serves as the loading control. **E.** Immunofluorescence (IFA) analysis with anti-HA antibody shows that DPAP3 is only expressed late at the schizont stage when the protein RAMA has already trafficked to the rhoptry organelle. Scale bar represents 5 μm.

Blood-stage *P*. *falciparum* strain 3D7 was cultured continuously [21] and ring-stage parasites were transfected as previously described [22] with the plasmid pDPAP3-HAglmS. This plasmid was adapted from pPTEX150-HAglmS [23] and incorporated the last kilobase of *dpap3* coding sequence in place of the *ptex150* coding sequence. The *dpap3* targeting region was amplified from *P*. *falciparum* 3D7 genomic DNA using the oligonucleotides 394F and 395R (see S1 Table for oligonucleotide sequences). Transgenic parasites were selected with 2.5 nM WR99210 (Jacobus). Following three rounds of drug cycling in the presence or absence of WR99210 to select for integrants, PCR was used to verify integration had occurred using the oligonucleotides indicated in Fig 1. The parasite population was then cloned out by limiting dilution and clones that were positive for integration by PCR were analysed further by Western blot and immunofluorescence analysis.

### Western blot analysis

*P*. *falciparum* infected RBC cultures were pelleted by centrifugation at 1500 *g* for 5 mins and parasites were harvested by treatment with 0.05% (w/v) saponin (Sigma). Parasites pellets obtained after centrifugation were washed with PBS containing cOmplete proteases inhibitors (Roche). The culture supernatants were centrifuged at 16,000 g for 30 min at 4°C to collect the merozoite pellet and an Amicon 10K centrifugal device (Merck Millipore) was used to concentrate the culture supernatant. Western blots of parasite proteins fractionated in 8% Bis-Tris gels (Life Technologies) were blocked in 3% BSA in PBS and then incubated with the following antibodies: mouse anti-HA (1:1,000; Roche), rabbit anti-EXP2 (1:1000)[24], monoclonal mouse anti-RAP1 7H8/50 (1:1000)[25], rabbit anti-RAMA-D (1:1000)[26] or rabbit anti-SERA5 (1:1000)[27]. After washing, the membranes were probed with horseradish peroxidase-conjugated secondary antibodies (1:5000; Thermo Scientific) and detection was performed using the Clarity™ ECL Western blotting substrate (Biorad). Membranes were imaged using a LAS-4000 Luminescent Image Analyzer (Fujifilm) and densitometry of bands was performed using ImageJ software (NIH, version 1.46r). Experiments were performed on at least three separate occasions, with the densitometry of bands of interest expressed relative to the EXP2 loading control, with results analysed using a two-tailed student’s t-test.

### Indirect immunofluorescence analysis (IFA)

IFA was performed on thin smears of infected RBCs fixed with ice cold 90% acetone/10% methanol for 2 minutes. Cells were blocked in 1% (w/v) BSA/PBS for 1 hour. All antibody incubations were performed in 0.5% (w/v) BSA/PBS. Primary antibodies for *P*. *falciparum* were used at the following concentrations: rat anti-HA (1:100, Life Technologies), mouse anti-HA (1:250, Life Technologies), monoclonal mouse RAP1 (1:1000), rabbit anti-RhopH3 (1:250)(kind gift from Ross Coppel), rabbit anti-RAMA-D (1:1000), rabbit anti-AMA1 (1:300)(kind gift from James Beeson), rabbit anti-RON6 (1:300)(kind gift from Ross Coppel), mouse anti-RESA (1:1000)(kind gift from Robin Anders) and mouse anti-MSP1_19_ mAb 17B6 (20μg/mL)[28]. After a one-hour incubation in primary antibody, cells were washed three times in PBS and incubated with the appropriate AlexaFluor 488/568-conjugated secondary antibodies (1:2000) for 1 hour. Cells were washed three times in PBS, and mounted with Prolong Gold Antifade reagent (Life Technologies) containing 4',6-diamidino-2-phenylindole (DAPI) (VectorLabs). Images were taken on an Olympus IX71 microscope and processed using ImageJ v1.46r.

### Immunoelectron microscopy

Magnetically-purified *P*. *falciparum* D10 and *Pf*DPAP3-HAglmS parasites at late schizont stage were fixed with 1% (v/v) glutaraldehyde for 30 min on ice. Fixed cells were pelleted (3,000 g for 1 min) and washed 3 times in ice-cold PBS, equilibrated into water, deposited into low melting point agarose plugs for ease of handling, then dehydrated in series of increasing ethanol concentrations. Parasites were embedded in LR Gold resin (ProSciTech) that was polymerized with benzoyl peroxide (SPI-Chem). Ultrathin 100 nm sections were cut with an Ultracut R ultramicrotome (Leica) and labeled with anti-HA mAb (0.7 μg/ml 12CA5, Roche) followed by goat-anti-mouse IgG conjugated to 12-nm gold (diluted 1:20, Jackson ImmunoResearch). The sections were then post-stained with uranyl acetate and lead citrate and observed at 120 kV on a CM120 BioTWIN transmission electron microscope (Philips).

### Knockdown and analysis of DPAP3 expression levels

Cultures of RBCs infected with ring-stage PfDPAP3-HAglmS parasites or the parental 3D7 line were cultured in the presence of 0.3 mM or 2.5 mM glucosamine (GlcN) or vehicle control (DMSO, 0 mM GlcN). Parasites were harvested at schizont stage in the same cycle or in the following cycle. Expression of DPAP3 and EXP2 was measured by Western blot analysis, with expression of DPAP3 normalised to EXP2 expression. Experiments were performed on three separate occasions.

### Analysis of parasite growth and egress

*P*. *falciparum* cultures were synchronized with 5% sorbitol and the invasion window kept to within 4 h by treating parasite cultures with 30 IU/ml heparin (Provet). After invasion, the synchronized cultures of *P*. *falciparum* 3D7 parental parasites or PfDPAP3-HAglmS parasites were washed three times with RPMI-1640 to remove heparin and treated with 2.5 mM GlcN at ring stage or left untreated (0 mM GlcN). Parasitemias in Giemsa-stained smears were determined by counting a minimum of 1000 RBCs. Parasite growth was also assessed using a SYBR Green I-based growth assay. For this infected RBCs cultured in 96-well U-bottom plates were harvested at trophozoite stage for a total of four cycles and snap frozen at -80°C. Following thawing, lysis buffer (20 mM Tris pH 7.5, 5 mM EDTA, 0.008% saponin, 0.08% Triton X-100) containing 0.2μl/ml SYBR Green I was added to parasite cultures in a 1:1 ratio. After incubation in the dark for 1 h, fluorescence activity was measured on a plate reader using excitation and emission wavelengths of 485 nm and 528 nm, respectively. All data were corrected for the background fluorescence of uninfected RBCs.

To analyse merozoite egress from RBCs, cultures synchronized as above were returned to culture medium containing 30 IU/ml heparin and either 0 mM or 2.5 mM GlcN. When the parasites developed into schizonts, 2.5 μM Compound 1 (C1) [29]was added for a duration of 4 h to arrest parasite egress. The cultures were then washed three times with RPMI-1640 media to remove Compound 1 and incubated with fresh RBCs under shaking conditions (180 rpm) for 30 min to facilitate invasion. Samples were collected immediately and for up to 2 hrs later, fixed with 1% PFA and stained with 1 mM Hoechst 34580 prior to quantitation of the number of schizonts by flow cytometry.

### Real-time PCR

RNA was extracted from schizont stage parasites using Trisure (Bioline) and contaminating genomic DNA was removed by DNaseI treatment (Invitrogen). cDNA was prepared using the Omniscript RT Kit (Qiagen) in accordance with the manufacturer’s protocol. The resulting cDNA was used to determine expression levels of the gene of interest by quantitative real-time PCR. Pf18SrRNA was used as a control to allow comparison of gene expression between parasite lines. Real time PCR was performed using the SensiFAST TM SYBR Lo-ROX Kit (Bioline) using a three-stage thermal profile. After heating cDNA to 95°C for 2 mins, annealing and extension were performed for 40 cycles consisting of 95°C for 10s, 50–65°C for 20s followed by 60°C for 30 s. The final dissociation protocol used gradual heating from 55°C to 95°C, incrementing by 0.5°C/30 s; this was used to plot a melting curve to confirm the specificity of the primers and product formation. Control reactions in which reverse transcriptase or cDNA template had been excluded were performed with each experiment to exclude genomic DNA contamination. All reactions were performed on three separate RNA harvests, with reactions performed in triplicate. Relative gene expression was quantified using the 2^−ΔΔ^*C*_T_ method [30].

## Results

### Generation of PfDPAP3-HAglmS to assess localisation and function of DPAP3

In this study, we attempted to explore DPAP3 function using a reverse genetic approach. Considering the haploid nature of the genome of *Plasmodium* and the previous described role for DPAP3 that indicated a conventional knockout of DPAP3 may be lethal [10], we directly opted to generate a transgenic *P*. *falciparum* (Pf) DPAP3-HAglmS parasite line that allowed DPAP3 expression to be conditionally regulated. This was achieved by transfecting a targeting construct pDPAP3-HAglmS into the *P*. *falciparum* 3D7 strain. This targeting construct was designed to modify the endogenous *dpap3* gene by incorporating the GlcN inducible *glmS* ribozyme within the 3’ untranslated (UTR) region of *dpap3*, thereby leading to expression of a chimeric *dpap3-*ribozyme mRNA (Fig 1A). The degree of ribozyme self-cleavage of the chimeric mRNA and thus the level of gene knockdown can be controlled by adding various concentrations of glucosamine (GlcN) to the parasite culture medium. Monitoring of DPAP3 expression was facilitated by the in-frame fusion a triple haemagglutinin (HA) and streptavidin tag to the C-terminus of DPAP3 in PfDPAP3-HAglmS. Genomic DNA extracted from transgenic parasites were analysed by PCR, which confirmed the parasite population had integrated the targeting construct as expected (Fig 1B). The transgenic parasites were cloned and western blot of parasite lysates using anti-HA antibodies validated that the endogenous DPAP3 had been successfully epitope-tagged (Fig 1C).

### DPAP3 is an apical protein expressed during schizogony

As DPAP3 has not been characterised previously, we took advantage of the fact that DPAP3 in PfDPAP3-HAglmS had been tagged with HA epitopes that allowed examination of the expression pattern of DPAP3 throughout the asexual cycle by western blot using anti-HA antibodies (Fig 1D). These results revealed that DPAP3 is expressed late in the cell cycle in schizont stages as well as in free merozoites, the latter indicating the protease is not completely secreted before merozoite egress (Fig 1D). DPAP3 was also released into the culture supernatant during parasite egress from the host erythrocyte. In these parasite stages the anti-HA antibody consistently recognized a predominant protein band of apparent molecular mass of ~120 kDa (Fig 1D). This is in keeping with the predicted molecular mass of DPAP3 after signal sequence cleavage (112 kDa) combined with the appended epitope tags (~4.5 kDa). An additional band of apparent mass of 85 kDa was also observed (Fig 1D). However, given this 85 kDa band was only observed in the occasional western blot, it is suspected that this band represents a protein degradation product during parasite lysis rather than it being a product of proteolytic cleavage of the full-length protein *per se*. No bands were observed in parental 3D7 parasites with the anti-HA antibody as expected.

**Fig 2:**
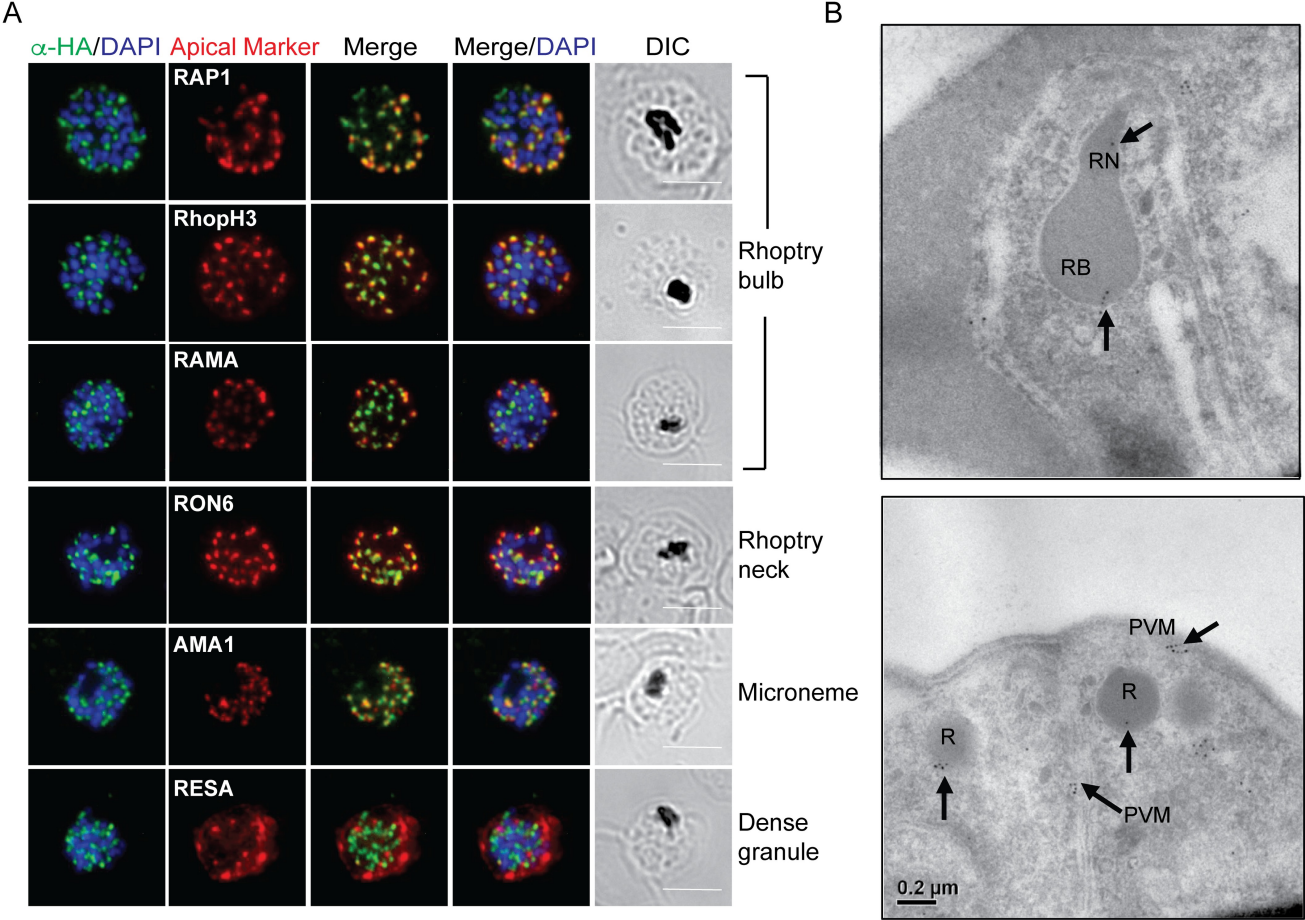
The localization of DPAP3 is distinct from proteins that localize to the apical organelles. **A.** IFA on RBCs infected with PfDPAP3-HAglmS parasites and fixed with acetone/methanol. DPAP3 is labeled with the anti-HA antibody. The scale bars represent 5 μm. **B.** Immuno-electron microscopy of PfDPAP3-HAglmS parasites with anti-HA antibody shows labelling for DPAP3 (as indicated by the arrowheads) in a mature schizont at the periphery of the rhoptry bulb (RB), rhoptry neck (RN) as well as at the PV/parasitophorous vacuole membrane (PVM).

Immunofluorescence analysis (IFA) with anti-HA antibodies was also performed to determine the localisation of PfDPAP3 in *P*. *falciparum* infected erythrocytes (Figs 1E and 2A). The results show a strong punctate pattern in the schizont stages, characteristic of apical localisation. No transient localisation characteristic of protein trafficking to the apical end of the parasite was observed for DPAP3 in earlier stage parasites and prior to when RAP1 is expressed. In an attempt to determine whether DPAP3 resides in a specific apical compartment, co-localisation studies were performed with antibodies to proteins that reside in apical organelles, namely RAMA, RAP1 and RhopH3, which reside in the rhoptry bulb; RON6, which resides in the rhoptry neck; AMA1, which resides in the micronemes and RESA, which localises to the dense granules. DPAP3 did not co-localise with any of these proteins (Fig 2A). Rather it showed partial co-localisation with rhoptry and microneme markers but no co-localisation with the dense granule marker RESA. This indicates that DPAP3 may not be housed in any of these three *Plasmodium* apical organelles, but potentially in secretory vesicles in close proximity to the rhoptries and micronemes.

To further examine the localisation of DPAP3, immunoelectron microscopy (immuno-EM) was performed using the anti-HA antibody. Whilst no substantial labeling was observed in wildtype parasites (S1 Fig), DPAP3 was observed at multiple locations in the parasites, including towards the periphery of rhoptry bulb and neck, in other apical regions and also at the parasitophorous vacuole membrane (PVM) (Fig 2B). Although the HA labelling seen in the immuno-EM was not obviously contained in membranous structures indicative of secretory vesicles, this is not surprising as the immuno-EM process used in this circumstance yields relatively poor membrane preservation.

### DPAP3 expression can be conditionally regulated

To investigate if DPAP3 expression could be regulated in response to GlcN, clonal lines of PfDPAP3-HAglmS parasites were synchronized and the following cycle, when parasites were at ring stages, cultured in the presence (+) or absence (-) of 0.3 mM or 2.5 mM GlcN. Protein samples were prepared from late schizont cultures within the same cycle (Cycle 1) or the following replication cycle (Cycle 2).

**Fig 3:**
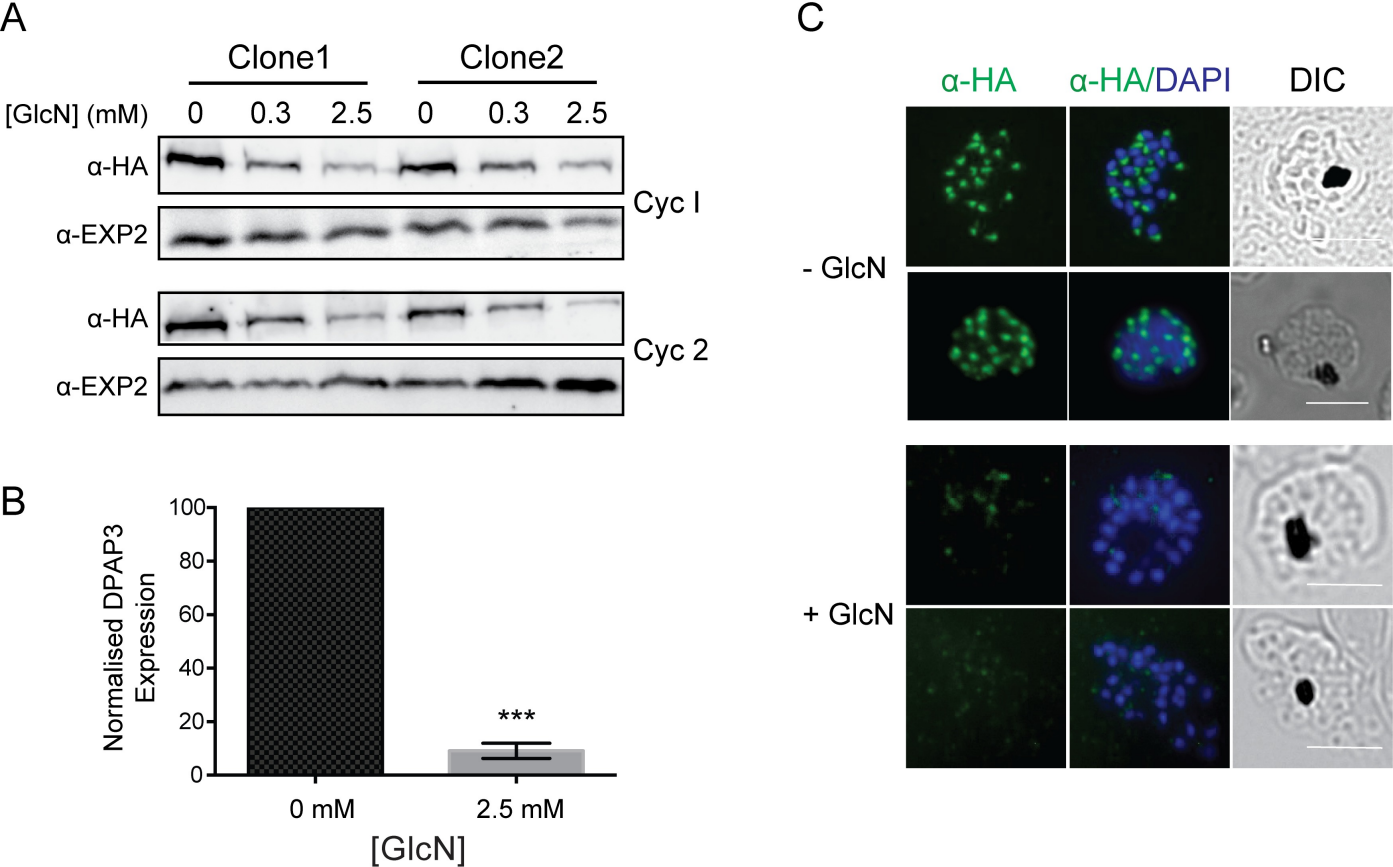
DPAP3 expression in *P*. *falciparum* can be conditionally regulated. **A.** Western blot analysis showing dose-dependent PfDPAP3-HA protein expression in two independent clones after treatment with glucosamine (GlcN) for one (upper panel) or two (lower panel) cell cycles (Cyc1 and Cyc2, respectively). EXP2 serves as the loading control. **B.** Densitometry of PfDPAP3 expression levels in PfDPAP3-HAglmS Clone 1 parasites grown in the presence or absence of glucosamine for one cycle revealed DPAP3 expression levels were knocked down by 91%. Error bars represent ± SEM from three independent experiments. **C.** IFA showing DPAP3-HA expression is significantly reduced within 24 hrs after addition of 2.5 mM GlcN when the parasites are at schizont stage. Scale bars represent 5 μm.

Addition of GlcN to PfDPAP3-HAglmS led to a dose-dependent expression of DPAP3 within 24 h (Fig 3A), with 91% knockdown of DPAP3 protein levels in the presence of 2.5 mM GlcN compared to the untreated control when normalised to expression of a control protein, EXP2 (Fig 3B). The response of clone 1 to GlcN was not significantly different from clone 2, hence all further analysis was performed on the PfDPAP3-HAglmS clone 1 parasites. DPAP3 knockdown at the protein level could also be observed in IFAs, where the +GlcN parasite schizonts showed only faint HA labelling compared to the bright punctate HA labelling observed in the–GlcN schizonts (Fig 3C).

### Effect of DPAP3 knockdown on the maturation and trafficking of apical proteins and SUB1 substrates

**Fig 4:**
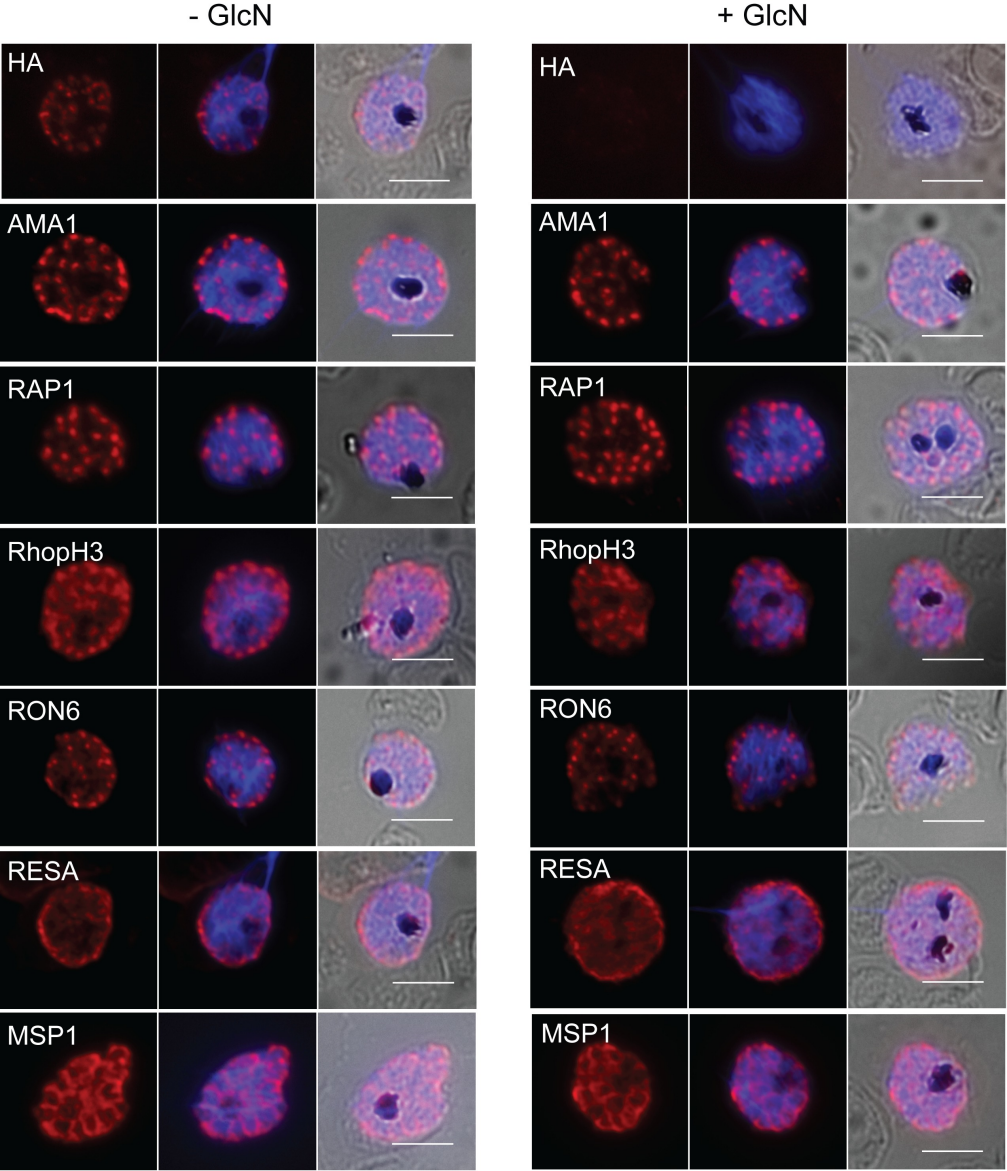
Effect of DPAP3 knockdown on the trafficking of apical proteins. IFA showing trafficking of selected proteins to the micronemes (AMA1), rhoptries (RAP1, RhopH3, RON6), dense granules (RESA) and merozoite surface (MSP1) is unaffected in the PfDPAP3-HAglmS parasites cultured in the presence of 2.5 mM glucosamine (GlcN). Scale bars represent 5 μm.

Since inhibition of DPAP3 has been shown to affect the localisation of the micronemal protein AMA1, we next investigated the impact of DPAP3 knockdown on the trafficking of several different apical proteins in the PfDPAP3-HAglmS line to establish whether DPAP3 is required for their maturation and correct trafficking to the respective organelles. Included in this analysis were AMA1 and known SUB1 substrates, MSP1 and RAP1. We also analysed the localisation of RhopH3, another rhoptry bulb protein predicted to be a SUB1 substrates but it has yet to be investigated whether processing of bulb proteins is critical for correct trafficking to this organelle. IFA showed that irrespective of the levels of expression of DPAP3, each of these apical proteins, as well the rhoptry neck protein RON6 and dense granule protein RESA included as controls could all traffic normally to their expected destination (Fig 4). This indicates that DPAP3 does not contribute to the trafficking of these proteins to their respective locations. Moreover, given that knockdown of DPAP3 expression did not impact on the ability or timing of the parasites to mature from rings into schizonts to perform this analysis, these results also indicate that DPAP3 is not required for the normal maturation of schizonts.

### DPAP3 knockdown does not affect the processing of SUB1 substrates

**Fig 5:**
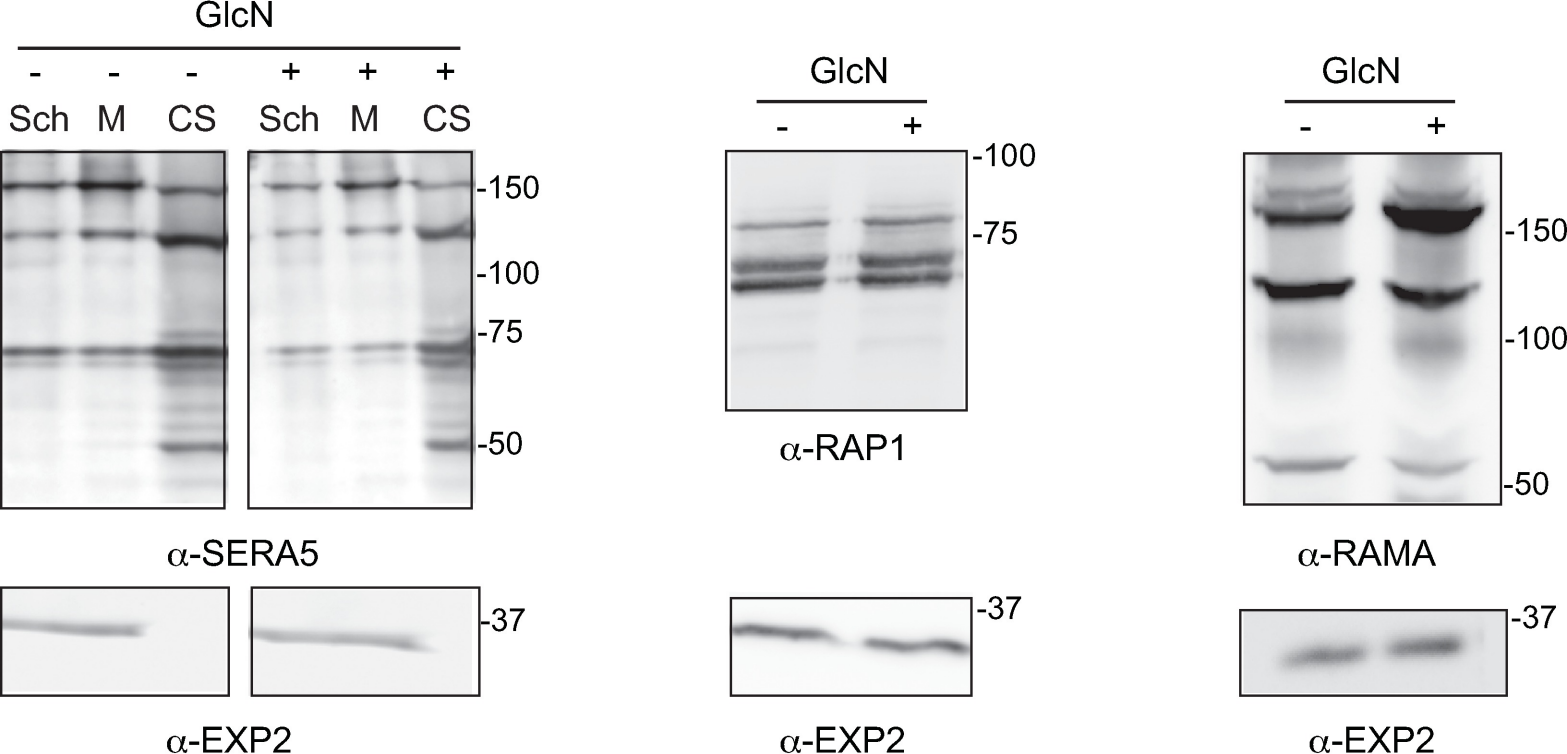
Knockdown of DPAP3 does not affect maturation of SUB1 substrates. Representative western blots (n = 3–5) of schizont (Sch) and merozoite (M) lysates and culture supernatant (CS) collected after egress (SERA5) or schizont lysates (RAP1, RAMA) made from infected RBCs grown in the presence (+) or absence (-) of 2.5 mM GlcN. Densitometry of bands relative to the EXP2 loading control and between treatment groups revealed no significant difference in the processing of SERA5, RAP1 and RAMA as a result of DPAP3 knockdown.

As the SAK1 inhibitor affects SUB1 maturation and hence SERA5 processing, we next examined whether knockdown of DPAP3 affects the maturation of the SUB1 substrates SERA5 and RAP1 as well RAMA, a predicted SUB1 substrate. During merozoite egress, SUB1 normally processes the 126 kDa PV-located protein SERA5 into a 73-kDa and 56-kDa form, the latter of which is then further processed in a SUB1-independent manner into a 50-kDa form [31]. In the case of RAP1, N-terminal truncation of the 84-kDa precursor form gives rise to an 82-kDa form, a 70-kDa intermediate form and a final processed product of 67 kDa. The last processing step is mediated by SUB1 and occurs late in schizont maturation [32,33]. It has been hypothesised that processing of RAP1 is required to release it from a complex that contains RAMA once it is trafficked to the rhoptries [34]. Interestingly the 170-kDa RAMA protein is processed into a 60-kDa form, and both an *in silico* prediction tool and proteomic analysis have flagged this protein as being processed by SUB1 [14]. However, in all cases densitometry analysis of western blots of PfDPAP3-HAglmS parasite lysates made from either schizont-infected RBCs, free merozoites or culture supernatant using antibodies to SERA5, RAP1 and RAMA (and EXP2 as a normalising control), showed there was no significant accumulation in any of the precursor forms of these proteins in the presence of GlcN (Fig 5). These results are, therefore, inconsistent with a role for DPAP3 in SUB1 maturation.

### DPAP3 knockdown does not affect parasite egress

**Fig 6:**
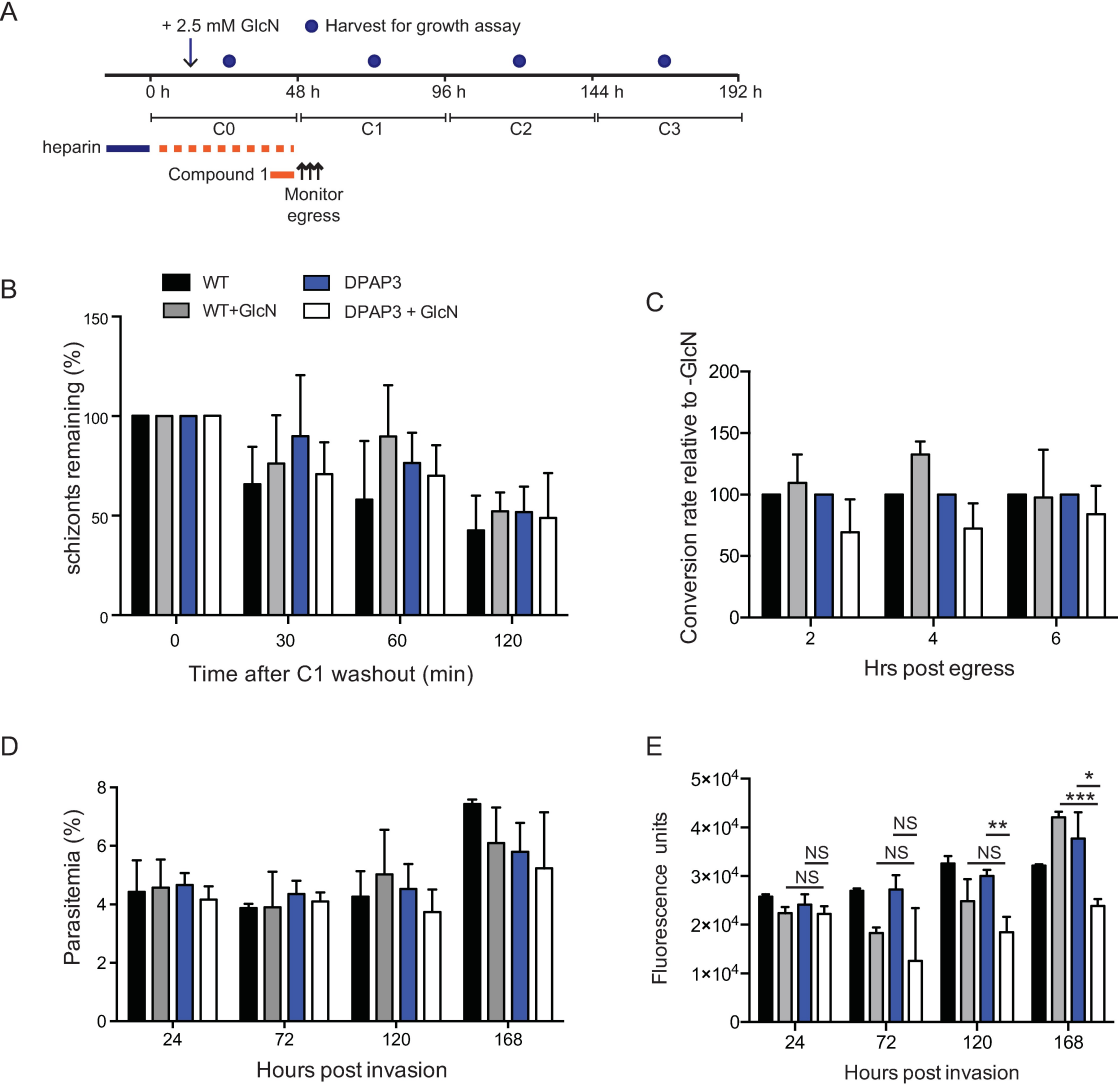
Knockdown of DPAP3 does not affect parasite egress or growth. **A.** Overview of egress and growth experiments. For both experiments, *P*. *falciparum* wildtype 3D7 (WT) and PfDPAP3-HAglmS lines were synchronised with heparin (blue line). Heparin was washed out to allow invasion to occur, after which 2.5 mM GlcN was added. For the egress experiments heparin was also added back to the culture medium (red dotted line), followed by the addition of Compound 1 for 4 hours (red line) when parasites reached schizogony. Egress was monitored at the indicated time points after heparin and Compound 1 were washed out. For the growth experiments, infected erythrocytes were harvested mid-cycle for analysis by microscopy and SYBR Green I assay. **B.** Analysis of the percentage of schizonts remaining at specified timepoints after Compound 1 washout reveal egress is unaffected by knockdown of DPAP3 expression (n = 3). **C**. Relative conversion rate of schizonts to rings, normalized to parasites grown in the absence of GlcN (n = 3) **D**. Analysis of parasite growth by microscopy. The parasitemias of cultures (diluted 1:10 each cycle) are plotted (n = 3). **E**. Analysis of parasite growth by SYBR Green I-based assay. Fluorescence units of cultures (diluted 1:10 each cycle) are plotted (n = 3). Results were analysed by student’s *t* test and comparison between PfDPAP3-HAglmS +/- GlcN or between PfDPAP3-HAglmS and parental 3D7 both grown in the presence of GlcN is indicated. NS, not significant, *p<0.05, **p<0.01, ***p<0.001.

DPAP3 has been previously implicated in parasite egress from RBCs and although the above findings suggest DPAP3 does not process SUB1, DPAP3 may be responsible for the processing of other parasite proteins that contribute to parasite egress. To examine this further, PfDPAP3-HAglmS or *P*. *falciparum* 3D7 parental parasites synchronised with heparin were grown in the presence or absence of GlcN as well as heparin and arrested at schizont stage with Compound 1 (C1) to ensure both groups were at the same developmental stage. Then, following heparin and C1 washout, egress was monitored and quantitated both by Giemsa stained blood smears and flow cytometry (see Fig 6A for overview of methodology). Knockdown of DPAP3 expression in the PfDPAP3-HAglmS parasite line did not lead to an accumulation of mature, unruptured schizonts. Indeed, quantitation of the number of schizonts remaining at particular time points post C1 washout revealed a similar rate of schizont reduction when compared to PfDPAP3-HAglmS parasites grown in the absence of GlcN such that by 120 min post C1 washout, ~50% of parasites had egressed irrespective of the parasite line and whether they had been exposed to GlcN treatment or not (Fig 6B). The conversion of schizonts to ring was also analysed (Fig 6C) and whilst knockdown of DPAP3 appeared to lead to a greater lag in invasion as indicated by fewer rings at the earlier time points compared to parasite lines expressing DPAP3, this was not statistically significant. The number of rings that had converted from the schizont populations by 6 h was also comparable between the lines.

To determine whether knockdown of DPAP3 had any longer term impact on parasite growth, we also monitored tightly synchronised PfDPAP3-HAglmS and parental parasites grown in the presence or absence of GlcN for a further three cycles as indicated in Fig 6A. Parasite cultures were kept below 5% parasitemia by splitting cultures 1:10 each cycle to prevent overgrowth. The parasitemia of cultures harvested approximately mid-cycle were measured by microscopy of Giemsa-stained blood smears and SYBR Green I-based assays that measure nuclear content were also conducted to measure parasite growth. While no significant reduction in parasitemia was observed over time when DPAP3 expression was knocked down (Fig 6D), the more sensitive SYBR Green I-based assay indicated that despite treatment with GlcN having some impact on parasite growth, including on the parental 3D7 line, knockdown of DPAP3 did ultimately have a significant effect on the parsaites after 168 h in culture (Fig 6E).

#### DPAP3 expression in not complemented by upregulation of other DPAPs

To confirm that the DPAP3 function was not completed by the upregulation of other DPAPs in PfDPAP3-HAglms parasites, reverse transcriptase quantitative PCR (RT-qPCR) was used to compare the transcript levels of *Pfdpap1* and *Pfdpap2* in parasites grown in the presence and absence of GlcN. The relative abundances of transcripts evaluated using the ΔΔ threshold cycle (CT) method using the constitutive 18S rRNA as the reference showed expression of *Pfdpap1* remained the same. As expected, the expression of *Pfdpap2* also remained unchanged in the knockdown parasites (S2 Fig).

## Discussion

Characterization of DPAP3 by epitope tagging this protein in *P*. *falciparum* revealed DPAP3 is expressed in schizonts and merozoite stages of the parasite lifecycle, thus coinciding with the timing of expression of apical proteins, including the rhoptry proteins. PfDPAP3 is consistently recognized on SDS-PAGE gels as a predominant protein band of apparent molecular mass of 120 kDa and appears to be secreted, given its presence in the culture supernatant following egress and its multiple localization as detected by immuno-EM–at the periphery of the rhoptries and the micronemes, as well as regions close to the PVM before invasion.

Knockdown of DPAP3 expression using GlcN led to substantially lower levels of the protein, however this did not impact on the ability of the parasites to mature into schizonts. Moreover, a selection of proteins that are normally trafficked via the secretory system to the micronemes (AMA1), or rhoptries (RAP1, RAMA, RhopH3, RON6) during schizogony all exhibited the expected localization profile after DPAP3 knockdown, indicating that DPAP3 is not required to correctly traffic these proteins during schizogony. This is in contrast to the inhibitor studies with SAK1 which revealed that inhibition of DPAP3 led to a complete loss of the precursor and intermediate forms of AMA1 and loss of AMA1 trafficking to the micronemes [10].

It has also previously been suggested that DPAP3 is responsible for the final processing step of SUB1, converting the 54-kDa form (that arises after self-processing of the 82-kDa precursor form) into a 47-kDa form. However, our analysis revealed that processing of SERA5, RAP1 and RAMA, which have been validated or predicted to be substrates of SUB1, were unaffected by the knockdown of DPAP3, arguing against a role for this protease in SUB1 maturation or as a general maturase for secreted proteins to facilitate trafficking to the apical organelles. That the aspartic protease plasmepsin X (PMX) has very recently been identified by as being responsible for the final SUB1 processing step such that its conditional knockdown leads to the accumulation of SERA5 and MSP1 precursor forms [35], is consistent with our findings that maturation of SUB1 substrates is not affected by DPAP3 knockdown. Moreover, Nasamu et al [35] also revealed that another aspartic protease termed PMIX, is responsible for the processing of the 84-kDa precursor form of RAP1, and consistent with this, localizes to the rhoptries [35]. Therefore, PMIX rather than DPAP3 may serve as the general maturase for secreted rhoptry proteins.

In keeping with a role for PMX in SUB1 maturation, this aspartic protease is also critical for parasite egress, with both PMIX and PMX essential for erythrocyte invasion [35]. On the other hand, our results indicate that DPAP3 does not play a role in parasite egress and the presence of DPAP3 in free merozoites is consistent with these findings. Although knockdown of DPAP3 affected longer-term growth of parasites in culture, we were not able to pinpoint a function for DPAP3. The timing of DPAP3 expression, its localization and secretion post-egress, together with a trend in DPAP3 knockdown parasites to take longer to invade RBCs, make it tempting to speculate that this protease may contribute to the maturation of proteins involved in RBC invasion. In the rodent malaria parasite, *P*. *berghei*, DPAP3 has been successfully knocked out in two separate studies without ablating parasite growth, which indicates this protease is not essential to parasite survival. Nevertheless knockout of DPAP3 in one study resulted in a relatively slower growth rate [20], and whilst no growth defect was observed for up to 5–7 days in another study, there was an impact in the ability of mice to succumb to cerebral malaria [19]. It is unlikely that DPAP3 expression could be complemented by DPAP1 as the latter localizes to the food vacuole and indeed we did not see a change in the expression of DPAP1 upon knockdown of DPAP3 in *P*. *falciparum*.

In conclusion, the findings from our studies in combination with the revelation that SUB1 is processed by PMX, all point to the inhibitor SAK1 exhibiting off-target effects that have led to DPAP3 being incorrectly implicated in SUB1 processing and parasite egress. Whether DPAP3 holds any promise as an antimalarial drug target remains to be established but the application of tools to conditionally knockout, rather than knockdown DPAP3 to potentially yield stronger phenotypes to pinpoint function may help in answering that question.

## Supporting information

S1 Fig Wildtype *P. falciparum* parasites do not label with anti-HA antibodies. Immuno-electron microscopy of *P*. *falciparum* D10 parasites labeled with anti-HA antibodies reveals no labeling.
(EPS)

S2 Fig Effect of *dpap3* knockdown on the expression of *dpap1*. Effect of *dpap3* knockdown on the relative expression of *dpap1* and *dpap2* at the transcriptional level in schizont stages by RT-PCR. Error bars represent ± SEM of three replicates from three independent experiments.
(EPS)

S1 Table List of oligonucleotides used in this study. (DOCX)
