# Supplementary material for: The cysteine protease dipeptidyl aminopeptidase 3 does not contribute to egress of Plasmodium falciparum from host red blood cells
Source: PLoS One. 2018 Mar 6;13(3):e0193538. doi: 10.1371/journal.pone.0193538 (PMC5839547; doi:10.1371/journal.pone.0193538)
Supplement: S1 Table — (DOCX) [file pone.0193538.s003.docx]

**S1 Table. List of oligonucleotides used in this study**

| **Oligonucleotide** | **Sequence (5’🡪3’)** | **Gene** |
| --- | --- | --- |
| f/394 | AGGAGATCTAATAACGAAATAAACATAAAG | *dpap3* |
| g/395 | TAGCTGCAGCTGTTTCTTTTTGTTTAACAAACAAG | *dpap3* |
| a/607 | CACCAAGGATGTAACGGTGGA | *dpap3* |
| b/276 | GTGATTTCTCTTTGTTCAAGGA | *glmS* |
| c/608 | AGATTGTGGTTCGTGTTATGCA | *dpap3* |
| d/MK51 | cctaggAGCGGCATAATCTGGAAC | *ha* |
| e/609 | TGCAGCTACATGAAGAAATAAGGCA | *dpap3* |
| 670 | GCTGACTACGtCCCTGCCC | *18SrRNA* |
| 671 | ACAATTCATCATATCTTTCAATCGGTA | *18SrRNA* |
| 637 | TGTTGAAGATTTCCCACACG | *dpap1* |
| 638 | TTCTTTACCCCAACCATTTCC | *dpap1* |
| 639 | TTTCCCACACGCTCGTAGAT | *dpap1* |
| 640 | CCAACCATTTCCCCAACTATT | *dpap1* |
| 641 | AAAACAAATCCGACGCAAAC | *dpap2* |
| 642 | TCCTTCCAATAGGAGGCAGA | *dpap2* |
| 643 | TGGGGATGTTTTTATGCTGAG | *dpap2* |
| 644 | CAGGTGCGTTTGTGTTGAT | *dpap2* |
